# Comprehensive Physicochemical Characterization and in Vitro Human Cell Culture Studies of an Innovative Biocompatible and Biodegradable Silk-Derived Protein Hydrolysate, SDP-4

**DOI:** 10.1021/acsomega.4c08514

**Published:** 2025-01-14

**Authors:** Hasham Shafi, Andrea J. Lora, Saurabh Aggarwal, David W. Infanger, Brian D. Lawrence, Heidi M. Mansour

**Affiliations:** † Florida International University Center for Translational Science, Port St. Lucie, Florida 34987, United States; ‡ Herbert Wertheim College of Medicine, Department of Cellular & Molecular Medicine, Florida International University, Miami, Florida 33199, United States; § Silk Technologies, Ltd., Maple Grove, Minnesota 55369, United States; ∥ Robert Stempel College of Public Health and Social Work, Department of Environmental Health Sciences, Florida International University, Miami, Florida 33174, United States; ⊥ College of Engineering and Computing, Department of Biomedical Engineering, Florida International University, Miami, Florida 33174, United States

## Abstract

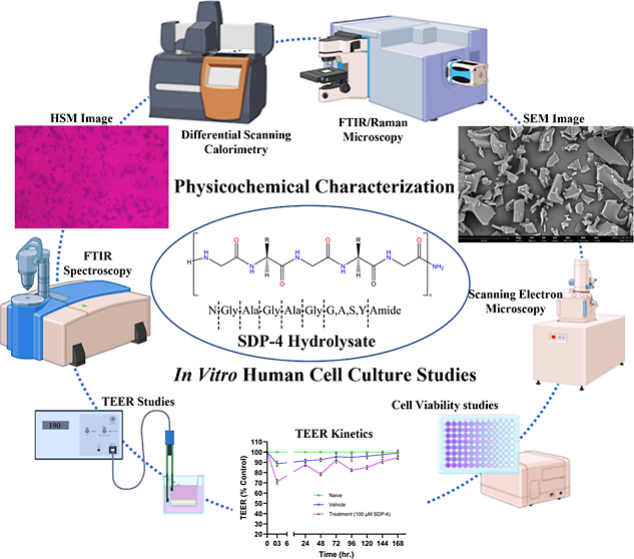

SDP-4 is a soluble silk fibroin-derived protein hydrolysate extracted from the *Bombyx mori* silkworm cocoon and is a novel first-in-class biopolymer that is biodegradable, biocompatible, and shown to have regenerative properties. SDP-4 is currently used as a commercial wetting agent in topical eye drops, but it has also been shown to have anti-inflammatory properties that could be utilized in other biomedical applications. The purpose of this study was to comprehensively characterize the physicochemical properties that are necessary to design formulations and examine cell viability in response to varying doses of SDP-4 on different human cell types, with a particular attention toward respiratory applications. Lyophilized SDP-4 powder was characterized by scanning electron microscopy (SEM), energy-dispersive X-ray (EDX) spectroscopy, differential scanning calorimetry (DSC), hot-stage microscopy (HSM), Karl Fisher (KF) coulometric titration, Raman spectrometry, confocal Raman microscopy (CRM), and Fourier transform infrared microscopy. The lyophilized powder exhibited a nonuniform, angular glassy flake morphology with uniform chemical composition and minimal moisture uptake when tested under varying humidity conditions. Crystalline character was evident through birefringence at ambient temperature which changed during phase transitions, as evidenced through qualitative and quantitative assessments. Dose ranging SDP-4 biocompatibility studies on different human lung cells, nasal cells, skin cells, and brain cells was assessed by the in vitro cell viability assay. Assay results showed that cell viability was maintained at the various doses studied for different human cell types. The transepithelial resistance (TEER) assay showed that SDP-4 leads to transient fluctuations in cell membrane integrity and barrier tightness, followed by a recovery phase as cells adapt or repair the junctions. These findings demonstrate that SDP-4 is biocompatible with different types of human cells and safe at all of the doses studied. The unique physicochemical properties of SDP-4 revealed in this study demonstrate its favorable formulating ability for a variety of potential therapeutic applications.

## Introduction

Therapeutic proteins have a wide range of applications in modern medicine in treating various conditions such as autoimmune diseases, cancer, genetic disorders, diabetes, etc., due to their unique pharmaceutical attributes like specificity, potency, and versatility.^1^ The field of therapeutic proteins has evolved significantly since the early 20th century, which began with the purification of insulin in 1922 for the treatment of diabetes. Current research continues to focus on enhancing stability, reducing immunogenicity, lowering production costs, and improving efficacy. Advances and innovations in protein engineering, personalized medicine, and delivery systems hold promise for development of novel protein-based therapies^2,3^

Fibroin protein, the central fiber component of the silkworm cocoon, is one of the most extensively studied natural biopolymer for use in biomedical applications today.^4^ Structurally, fibroin is a semicrystalline protein consisting of up to 55% beta-sheet regions within the natural silk fiber.^5^ The protein is primarily composed of heavy chain (390 kDa) and light chain (26 kDa) fragments connected together by a disulfide bond. There is also a small glycoprotein called P25 (30 kDa) that binds to both chains through noncovalent interactions to help maintain integrity of the fiber.^6^ The heavy chains have a highly repetitive hydrophobic amino acid sequence, that enable them to organize into β-sheet crystallites through hydrogen bonding.^7^ Aggregation and overall folding of the protein subunits gives rise to its ternary and quaternary structures.^8^

Fibroin can be readily extracted from silk cocoons using boiling alkaline water which will remove the sericin and P25 coating proteins, and the extracted fibers can be readily dissolved into water using heavy salt brines or ionic liquids to neutralize the extensive hydrogen bonding occurring between the beta-sheet structures.^9^ Processing salts or solvents can then be dialyzed away leaving fibroin dissolved in purified water, which can remain stable at refrigerated temperatures for several weeks before beginning to aggregate.^10,11^ Fibroin protein instability occurs as crystalline secondary structures begin to form and exclude water molecules from these hydrophobic regions as it is thermodynamically driven to self-assemble into water-insoluble aggregates.^12^ Biomedical engineers have learned to control and utilize this self-assembly process to form three-dimensional structures, such as films, sponge scaffolds, filaments, hydrogels, micronanoparticles, electro spun mats, and shaped Bioplastic objects.^10^ These material forms have been investigated for use in a variety of implementations in, wound healing, tissue engineering, medical devices, food coatings, bioelectronics, and drug delivery systems as more recent examples.^13−18^ In clinical practice, there are only a few commercialized fibroin products beyond classic silk sutures, which include SERI surgical scaffold, DermaSilk, Epifibroin 0039, and SilkVoice for applications in soft tissue repair, antibacterial dressing, wound healing dressing, and vocal fold medialization/insufficiency, respectively, with new products in clinical development as well.^13^

**Figure 1:**
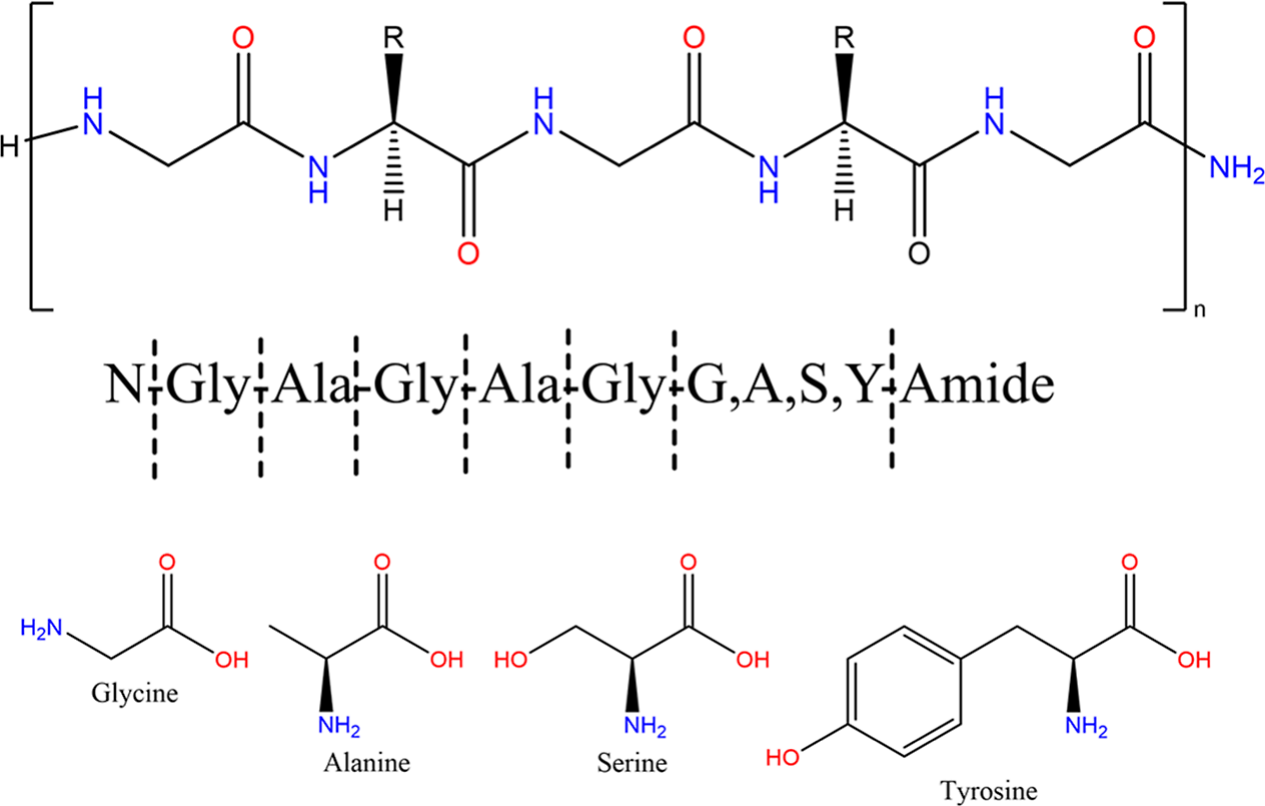
Repeating peptide sequence structure of raw SDP-4 powder and the amino acid composition. The chemical structures were drawn using ChemDraw ver. 21.0.0 (ChemOffice, Cambridge, MA, USA).

Despite the numerous potential medical applications highlighted in the literature, the use of regenerated fibroin protein is often limited by its unstable nature, leading to batch-to-batch inconsistency and limited shelf life in solution.^19^ One strategy to enhance the stability of fibroin is through hydrolysis processing, which can improve the solubility and bioavailability of the material by reducing the overall molecular size and modifying biochemical properties.^20,21^ One of these hydrolysates, silk-derived protein 4 (SDP-4), is composed of a repeating glycine–alanine repeating peptide motif that produces varying chain lengths with conserved amino acid content.^22^ SDP-4 has been recently utilized as a protein wetting agent for eye-drop formulations to treat a variety of ocular surface diseases by mimicking tear film mucin to provide lubrication, tear film stabilization, and promote corneal wound healing.^22−25^ The hydrolysis process results in creating a repeating peptide sequence of varying protein chain lengths, as shown in Figure 1. SDP-4 (average molecular weight of 20 kDa) differs biochemically to fibroin by having fewer disulfide bonds, fibroin light chain presence, and serine amino acid content that work to limit hydrogen bonding and beta-sheet formation between proteins to reduce the potential for aggregation.^22,26,27^

SDP-4 is a hydrophile and has a better solubility profile than the native fibroin protein due to its intrinsically disordered structure and reduced ability to form beta-sheets.^22^ Studies have shown that SDP-4 has dose-dependent anti-inflammatory properties by modulating the nuclear factor-kappa B (NF-kB) signaling pathway, thereby inhibiting proinflammatory mediators including TNF-α and MMP-9.^22^ Waleed et al. have shown that SDP-4 enhances the migration, adhesion, and proliferation of corneal epithelial cells in vitro using a scratch wound assay.^23^ A stable ophthalmic formulation of this protein has been demonstrated to accelerate wound healing on rabbit corneal epithelial surfaces.^25^ Recent clinical trials (NCT03889886 and NCT04535947) on a preservative-free eye drop formulation of this protein have shown that it is nonimmunogenic, well tolerated, actively absorbed from the ocular surfaces, and has rapid clearance from the blood.^24^ The study was able to show that SDP-4 ophthalmic solution improved comfort and tear film stability by acting as a mucin replacement and reduced dry eye symptoms.

This study reports on a comprehensive characterization of the physicochemical properties that are necessary for designing formulations and examines in vitro human cell viability in response to SDP-4 doses on different types of human cells. In addition, in vitro transepithelial electrical resistance of human cells was conducted to study the effects on the membrane. Surface morphology, thermotropic phase transitions, and molecular fingerprinting using various spectroscopy techniques were also conducted. The dose-cell response of SDP-4 on human cell viability on human nasal, skin, lung, and brain cells were quantified. The effects on the integrity and function of epithelial barriers were probed. These results establish a basis for further exploration of silk-derived proteins in drug delivery and tissue engineering. Ultimately, this work underscores the significance of advanced characterization methods in developing innovative biomaterials. As far as authors are aware, this is the first study to report such findings for SDP-4.

## Experimental Section

### Materials

Lyophilized SDP-4 (average *M*_W_ 20 kDa) was generously provided by Silk Technologies, Ltd. (MN, USA). Methanol of the HPLC grade was obtained from Fisher Scientific (Fair Lawn, NJ, USA), and ultrahigh purity (UHP) nitrogen gas was supplied by Airgas (Randor, PA, USA). Hydranal-Coulomat AD reagent was purchased from Honeywell Fluka (St. Louis, MO, USA), while anhydrous methanol was acquired from Millipore Corporation (MA, USA). Throughout the study, ultrapure water obtained from the Milli-Q P-QOD setup by Millipore (Fair Lawn, NJ, USA) was utilized.

A549 (adenocarcinoma human alveolar basal epithelial cells, deep lung respiratory region), H358 (bronchioalveolar human carcinoma epithelial cells, small airways respiratory region), Calu-3 (adenocarcinoma human lung bronchial epithelial cells, large airway conducting zone), RPMI 2650 (human nasal epithelial cells), and Eagle’s Minimum Essential Medium (EMEM) were obtained from ATCC (Manassas, VA, USA). Immortalized human keratinocytes (HaCaT) were sourced from AddexBio (San Diego, CA, USA). NHEKs Primary normal human epidermal keratinocytes (NHEKs), Normal Human Astrocytes (NHA, CC-2565), KGM Gold Keratinocyte Growth Medium BulletKit, AGM Astrocyte growth medium BulletKit Kit, and EGM-2 MV Microvascular Endothelial Cell Growth Medium-2 BulletKit were sourced from Lonza Walkersville, MD, USA. Human cerebral microvasculature endothelial cells (hCMEC/D3) were sourced from Merk, Darmstadt, Germany.

Rat tail Collagen I, Advanced Dulbecco’s Modified Eagle’s Medium (ADMEM), and Fetal Bovine Serum (FBS) were acquired from Gibco. Essential cell culture materials, including penicillin–streptomycin (10,000 U/mL) and amphotericin B (Fungizone), along with 96-Well Black/Clear Bottom Plates and Falcon Tissue Culture T75 Flasks, were sourced from Thermo Fisher Scientific (Miami, FL, USA). Transwell costar plates (12 well, 12 mm inserts, 0.4 μm polyester membrane) were procured from Corning, Fisher Scientific, Suwanee, GA, USA.

Furthermore, resazurin sodium salt was obtained from Acros Organics (NJ, USA), while dimethyl sulfoxide (DMSO) was acquired from Millipore-Sigma (St. Louis, MO, USA).

### Scanning Electron Microscopy

The surface morphology of the raw SDP-4 powder was examined using a ThermoFisher Phenom ProX G6 (NanoScience Instruments, ThermoScientific Instruments, Phoenix, AZ, USA), following protocols described in earlier studies.^28−31^ For sample preparation, powder samples were adhered to aluminum stubs with double-sided adhesive carbon tape (Ted Pella, Inc., Redding, CA, USA). A thin platinum alloy coating (∼7 nm thick) was applied using a Luxor Platinum sputter coater (NanoScience Instruments, Phoenix, AZ, USA) under argon plasma conditions (Airgas, Air Liquide, FL, USA). SEM images were captured with a Secondary Electron Detector at various magnifications using an accelerating voltage of 10 kV and a working distance of 7–8 mm. Image intensity adjustments were made using Phenom ProX G6 software (NanoScience Instruments, Phoenix, AZ, USA).

### Energy-Dispersive X-ray Spectroscopy

To analyze the elemental composition and identify potential impurities in the raw SDP-4, EDX was performed using the ThermoFisher Phenom ProX G6 (NanoScience Instruments, Phoenix, AZ, USA) at an accelerating voltage of 15 kV. The spot size was carefully adjusted to achieve a dead time of 20–30 s.

### Differential Scanning Calorimetry (DSC)

To study thermal properties and phase transitions of raw SDP-4 powder, a TA Discovery DSC250 (TA Instruments, New Castle, DE, USA) with T-Zero technology and an automated RSC-90 cooling system was used, following established protocols.^28−30,32,33^ Samples (1–2 mg) were carefully placed in hermetically sealed T-Zero aluminum pans with an empty sealed pan serving as the reference. UHP nitrogen gas was used as the purging gas at a flow rate of 50 mL/min. The samples were heated from 0.00 to 400.00 °C at a rate of 5.00 °C/min. All experiments were performed in triplicate, and the data were analyzed by using TRIOS software.

### Thermal Analysis Using Cross-Polarized HSM

A Leica polarized microscope (Leica DMLP, Wetzlar, Germany) equipped with a Mettler FP82 Hot Stage (Mettler-Toledo, LLC, OH, USA) was used to study the phase transitions of raw SDP-4 powder, following previously published protocols.^28−30^ A γ530 nm U-TP530 filter (Olympus, PA, USA) was used for light filtration. The powder samples were placed on glass slides, attached to the hot stage, and heated from 20.0 to 300.0 °C at a rate of 5.0 °C/min. Images were captured using a Nikon Digital Sight 1000 camera with 10× optical zoom, operated through Nikon NIS Elements software (Nikon Coolpix 8800, Nikon, Tokyo, Japan).

### Raman Spectral Analysis

The raw SDP-4 powder was analyzed using a DXR Raman system (Thermo Scientific, Fitchburg, WI, USA) with a 785 nm diode laser at 30 mW intensity, adhering to established protocols.^29,30,33,34^ Raman spectra, averaged over three measurements, were collected using 16 sample exposures of 4 s each. The confocal hole was set to 50 μm, and a grating of 400 lines/mm was used. Baseline correction and smoothing were applied, and data were processed using OMNIC (v9.12.1019) and OMNIC Atlμs software (v9.12.990).

### Confocal Raman Microscopy (CRM)

Raman imaging maps of raw SDP-4 powders were meticulously generated by using a DXR Raman system (Thermo Scientific, Fitchburg, WI, USA) paired with an Olympus BX41 confocal optical microscope (Olympus America, Inc., Chester Valley, PA, USA). A 10× objective and10 μm *X*–*Y* steps generated a grid of 9 Raman acquisitions, each based on 16 exposures of 4 s. Baseline corrections ensured spectral accuracy, and data were analyzed using OMNIC for Dispersive Raman (v9.12.1019) and OMNIC Atlμs (v9.12.990) software, revealing the spatial distribution of Raman-active species in the SDP-4 sample.

### ATR-FTIR Spectral Analysis

The ATR-FTIR spectra of raw SDP-4 powder were recorded using a Nicolet iS50 FTIR Spectrometer (ThermoScientific, USA) over a wavelength range of 4000–700 cm^–1^. Each measurement was performed in triplicate with background spectra acquired under identical conditions. Data analysis, including baseline correction and smoothing, was conducted using OMNIC (v9.12.928) and OMNIC Atlμs (v9.12.990). The experimental setup followed previously reported protocols.^29,35−37^

### Infrared (FTIR) Microscopy Analysis

The raw SDP-4 powder was analyzed for molecular fingerprinting using a Nicolet Continuum Infrared Microscope (ThermoScientific, USA), equipped with a mercury–cadmium–telluride detector. Spectra were obtained using a 15× objective, capturing 9 acquisitions at 10 μm intervals in the *X* and *Y* directions. The spectra (scanned 32 times) were obtained within a range of 4000–700 cm^–1^. Data analysis involved background subtraction, baseline correction, and smoothing using OMNIC software (v9.12.928 and v9.12.990).

### Residual Water Analysis Using Karl Fisher Coulometric Titration

Residual water content in the SDP-4 powder was assessed using KF Coulometric titration, following previous lab protocols.^29,30,33,38^ Titration was carried out using a TitroLine 7500 KF trace titrator (SI analytics, Weilheim, Germany). A precise amount of 2–3 mg of powder was weighed and placed in the reaction cell containing Hydranal Coulomat AD reagent (Honeywell Fluka, Seelze, Germany). The water content was determined by averaging the results from three samples.

### Water Vapor Sorption Isotherm Analysis

Water vapor sorption isotherms for SDP-4 powder was measured using a Discovery SA-Dynamic Vapor Sorption Analyzer (Waters, TA Instruments, New Castle, DE, USA), adhering to established protocols.^39,40^ Approximately 1.0–2.0 mg of raw SDP-4 powder was dried at 25 °C and 0.0% relative humidity (RH) for up to 7 h. The samples were then exposed to increasing RH levels from 0.0% to 95% RH in 5% increments, with each step lasting 3 h. Equilibrium was achieved when the weight change was ≤0.03% (w/w).

### Dose-Dependent In Vitro Cell Viability

Resazurin Assay was performed to access cell viability of the various concentrations of raw SDP-4 lyophilized powders on A549 (passage number 13), H358 (passage number 4), Calu-3 (passage number 8), and HaCaT (passage number 9). NHEK (passage number 6), RPMI 2650 (passage number 7), NHA (passage number 4), and hCMEC/D3 (passage number 34) according to previously published protocols.^34,36,37,40−43^ A549, H358, and HaCaT cells were cultured in T-75 flasks (collagen-coated) using ADMEM, supplemented with 10% (v/v) FBS, fungizone (0.5 μg/mL amphotericin B, 0.41 μg/mL sodium deoxycholate), 2 mM l-glutamine, and Pen–Strep (100 units/mL penicillin, 100 μg/mL streptomycin) at 37 °C with 5% CO_2_ in a humidified incubator. Similarly, Calu-3 and RPMI 2650 cells were cultured in EMEM by using the same supplements.

NHEK cells were grown in T-75 flasks using KGM Gold Keratinocyte Growth Medium BulletKit, which was prepared by combining the KGM Gold SingleQuots supplements with the KBM Gold Basal Medium (nonsupplemented) as per the manufacturer’s guidelines. The cells were maintained in a humidified environment at 37 °C with 5% CO_2_.

NHA cells were cultured in T-75 flasks using AGM Astrocyte Growth Medium BulletKit, which was prepared by combining AGM SingleQuots Supplements to the ABM Basal Medium (nonsupplemented media) following the manufacturer’s guidelines. The cells were incubated in a humidified environment at 37 °C with 5% CO_2_.

hCMEC/D3 cells were grown in T-75 flasks using EGM-2 MV Microvascular Endothelial Cell Growth Medium-2 BulletKit, prepared by adding the EGM-2 SingleQuots Supplements to the nonsupplemented EBM-2 Basal Medium, following manufacturer’s instructions. The cells were incubated in a humidified incubator at 37 °C with 5% CO_2_.

To access viability, cells were harvested at approximately 80% confluency using trypsin–EDTA solution at 37 °C and then plated into 96-well black plates at a density of 5000 cells per well in 100 μL of medium. After a 48 h incubation period to allow for attachment and monolayer formation, cells were treated with different concentrations of lyophilized SDP-4 solution (0.1, 1, 10, 100, and 500 μM). The SDP-4 solutions were prepared by dissolving SDP-4 powder in nonsupplemented media specific to the cell type. Each well was treated with 100 μL of either the SDP-4 solution or the control (Non-Supplemented Media).

After 48 h of treatment at 37 °C and 5% CO_2_, 100 μL of resazurin sodium salt solution (20 μM, prepared in nonsupplemented media) was added to each well and incubated for an additional 4 h. The reduction of resazurin to resorufin by viable cells was quantified by measuring fluorescence at 544 nm excitation and 590 nm emission using the Synergy H1Multi-Mode Reader (BioTek Instruments, Inc., Winooski, VT, USA). The cell viability was calculated based on the resulting fluorescence signals using eq 1:
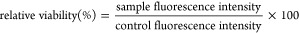
1

### In Vitro TEER Assessment of Skin Epithelial Cells under the Air–Liquid Interface

TEER measurements were performed to evaluate the barrier integrity, tightness, and recovery of HaCaT cells following SDP-4 treatment according to our previously published protocols.^31,37,40,43^ Cells were cultured in ADMEM supplemented with 10% FBS, Pen–Strep (100 U/mL penicillin and 100 μg/mL streptomycin), and fungizone (0.5 μg/mL amphotericin B, 0.41 μg/mL sodium deoxycholate) at 37 °C with 5% CO_2_. Upon confluence, the cells were seeded (500,000 cells/well) onto Costar Transwell inserts (0.4 μm polyester membrane, 12 mm for a 12-well plate) with media added apically (0.5 mL) and basolaterally (1.5 mL). Media was replaced every other day from the basolateral side.

After ∼7 days, a dense monolayer formed, with TEER values stabilizing at ∼190 Ω cm^2^. TEER values were recorded using an STX4 open-end electrode connected to the EVOM Manual TEER measurement meter (World Precision Instruments, Sarasota, Florida, USA). Media was removed from the apical side to establish air–liquid interface (ALI) conditions, stabilizing TEER values around 170 Ω cm^2^ after 72 h. The cells were treated with 100 μM SDP-4 solution prepared in nonsupplemented EMEM, and TEER values were recorded after 3 h and then after every 24 h for 7 days using an STX4 open-end electrode. Naïve and vehicle-treated cells were included as controls. TEER measurements involved replacing basolateral media with 1.5 mL of PBS and adding 0.5 mL of PBS apically. Postmeasurement, cells were immediately returned to ALI with fresh basolateral media. TEER kinetics for naïve and vehicle-treated cells (*n* = 4 replicates) were plotted in GraphPad Prism v10.2.3 (GraphPad Software, Inc., Boston, MA) as a percentage of control using eq 2
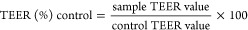
2

## Results

### Morphological Analysis by SEM

**Figure 2:**
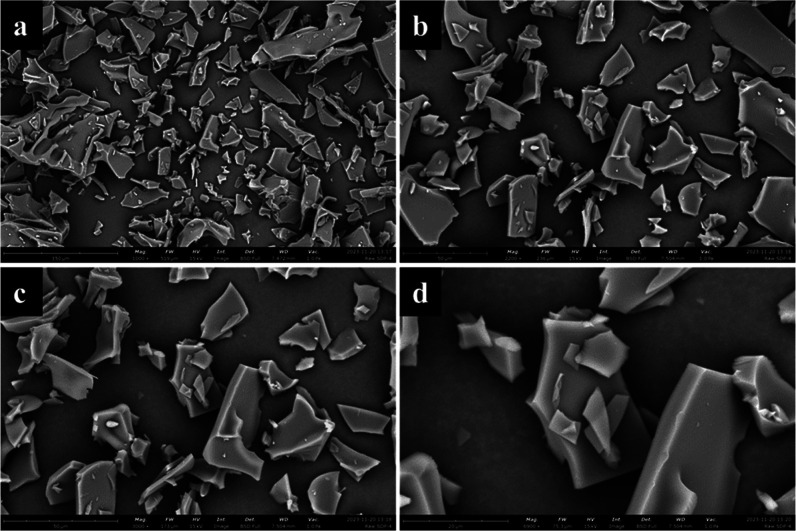
SEM images illustrating the surface morphology of raw SDP-4 powder captured at varying magnifications (a) 1000×, (b) 2200×, (c) 3000×, and (d) 6900×.

Figure 2 shows SEM micrographs of raw SDP-4 particles at different magnifications, revealing an angular, smooth, and glassy flake morphology. Particle size analysis conducted with ImageJ software (U.S. NIH, Maryland, USA) revealed a broad size distribution with a mean geometric diameter of 19.22 ± 15.08 μm and a range of 2.17–114.55 μm.

### Elemental Fingerprinting by SEM–EDX

**Figure 3:**
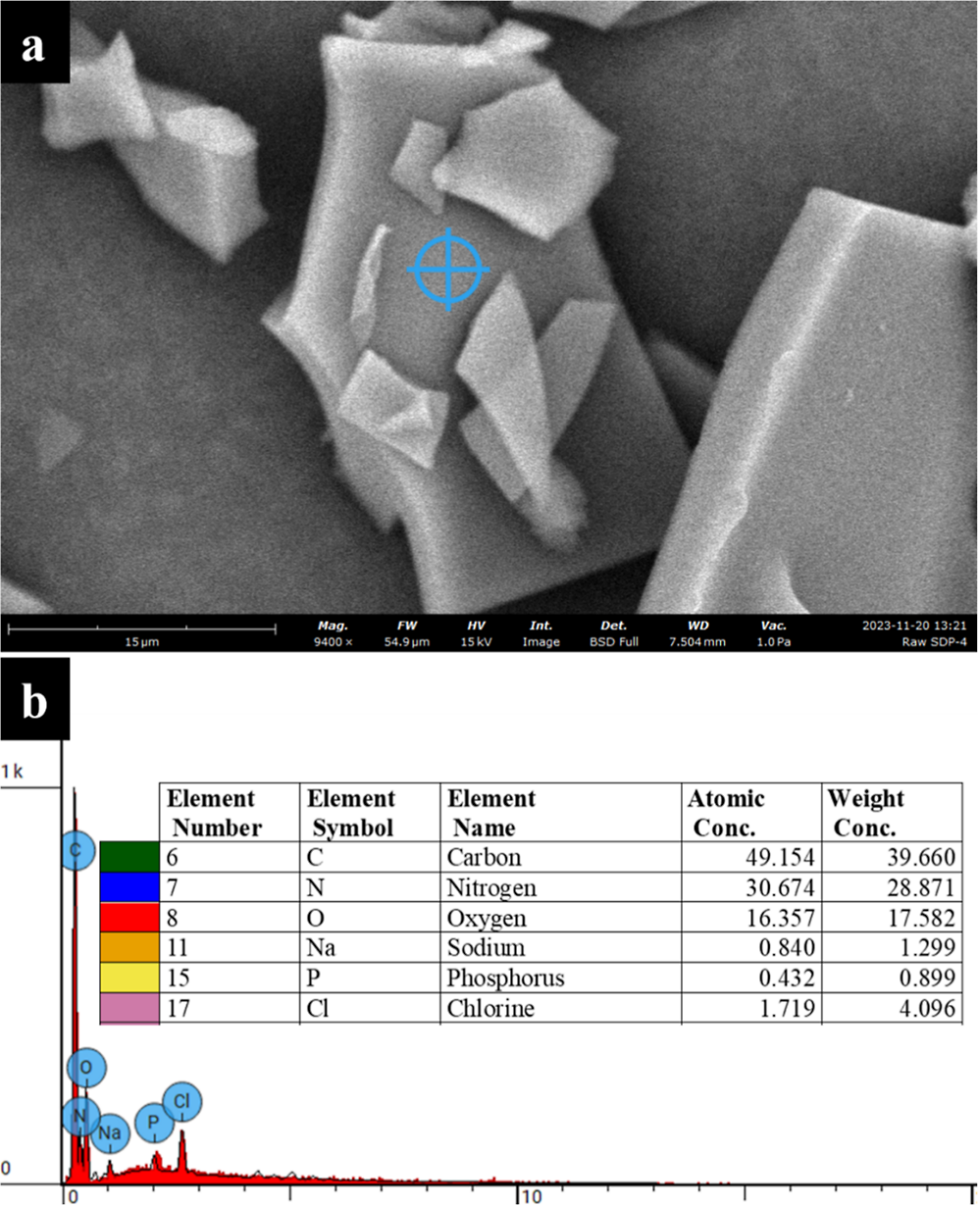
(a) Representative SEM micrograph taken at 9400×, showing the elemental spot analysis of the surface of raw SDP-4 particle via EDX spectroscopy. (b) Corresponding EDX spectrum displaying the elemental components identified in the SDP-4 particle. The inset table provides the atomic and weight percentages of the detected elements.

Figure 3 displays the elemental fingerprinting of raw SDP-4 particles, indicating the occurrence of C, N, O, Na, Cl, and P. The elements C, N, and O are present in higher proportions, forming the structural components of amino acids in SDP-4. Minor peaks of Na, Cl, and P indicate residues from the processing and purification of silk fibroin. Trace quantities of P suggest post-translational modification of the silk fibroin protein. According to the United States Pharmacopeia (USP), limits for impurities, particularly heavy elements, are based on permitted daily exposure. The impurities reported here are not heavy elements and are commonly present in body fluids.

### Phase Transition Behavior by DSC

**Figure 4:**
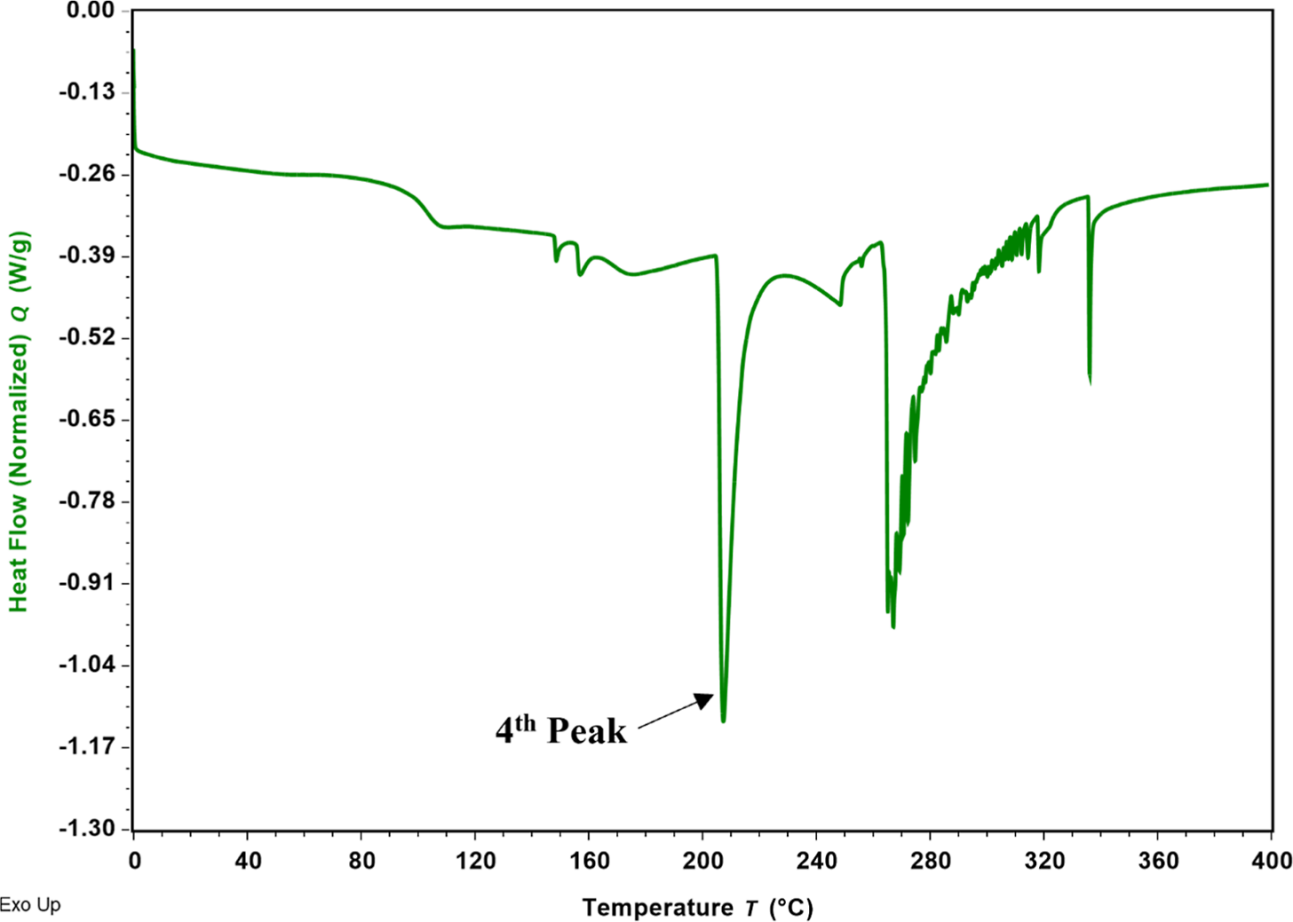
Representative DSC thermogram of raw SDP-4 powder showing thermal transitions.

Figure 4 represents the DSC thermogram of the raw SDP-4 powder, and Table 1 shows the main thermodynamic transitions calculated by using TRIOS software, revealing key thermal properties of the protein. The primary thermal transition, observed at a peak temperature of 216.79 ± 10.83 °C with an enthalpy of 49.17 ± 3.55 J/g, corresponds to the melting point of the SDP-4 powder. Additionally, the glass transition temperature (*T*_g_) of the SDP-4 powder was determined to be 103.54 ± 1.23 °C using the inflection midpoint method.

**Table 1:** Thermal Analysis of the Raw SDP-4 Powder by DSC, Presented as Mean ± SD (*n* = 3)

peak position	1st peak	2nd peak	3rd peak	4th peak	5th peak
enthalpy (J/g)	6.35 ± 1.02	0.81 ± 0.12	1.95 ± 0.60	49.17 ± 3.55	6.60 ± 3.42
peak temperature (°C)	109.22 ± 4.53	152.09 ± 5.11	159.31 ± 2.91	216.79 ± 10.83	242.48 ± 8.87

### Phase Transitions under HSM Cross-Polarizers

**Figure 5:**
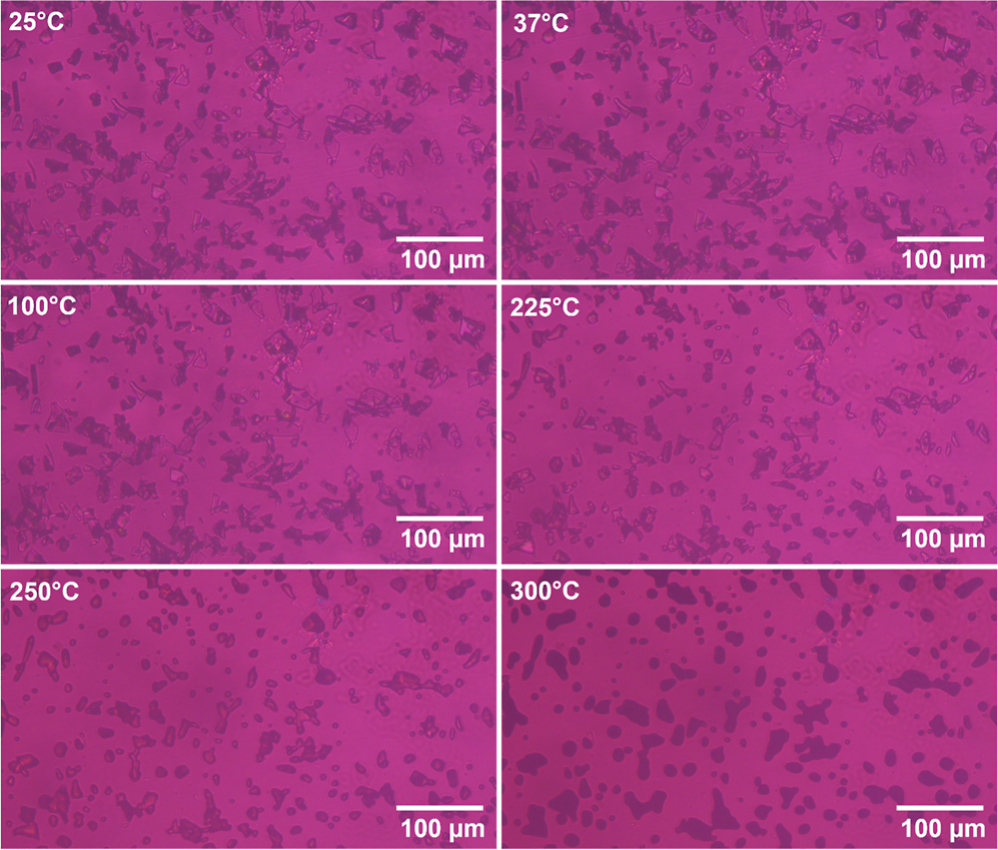
Representative HSM images of raw SDP-4 taken (scale 100 μm) at different temperatures.

The HSM images of raw SDP- 4 taken at different temperatures are shown in Figure 5. At temperatures below its melting point (216.79 ± 10.83 °C), as reported above in the DSC, there is a presence of a certain degree of birefringence, suggesting the semicrystalline nature of the protein. Above the melting point, the protein’s crystalline regions transition into a liquid, and consequently, its birefringence is lost. As the temperature increases further, the protein undergoes significant thermal degradation, resulting in the elimination of the ordered structure and complete loss of birefringence.

### Structural Analysis by Raman Spectroscopy

**Figure 6:**
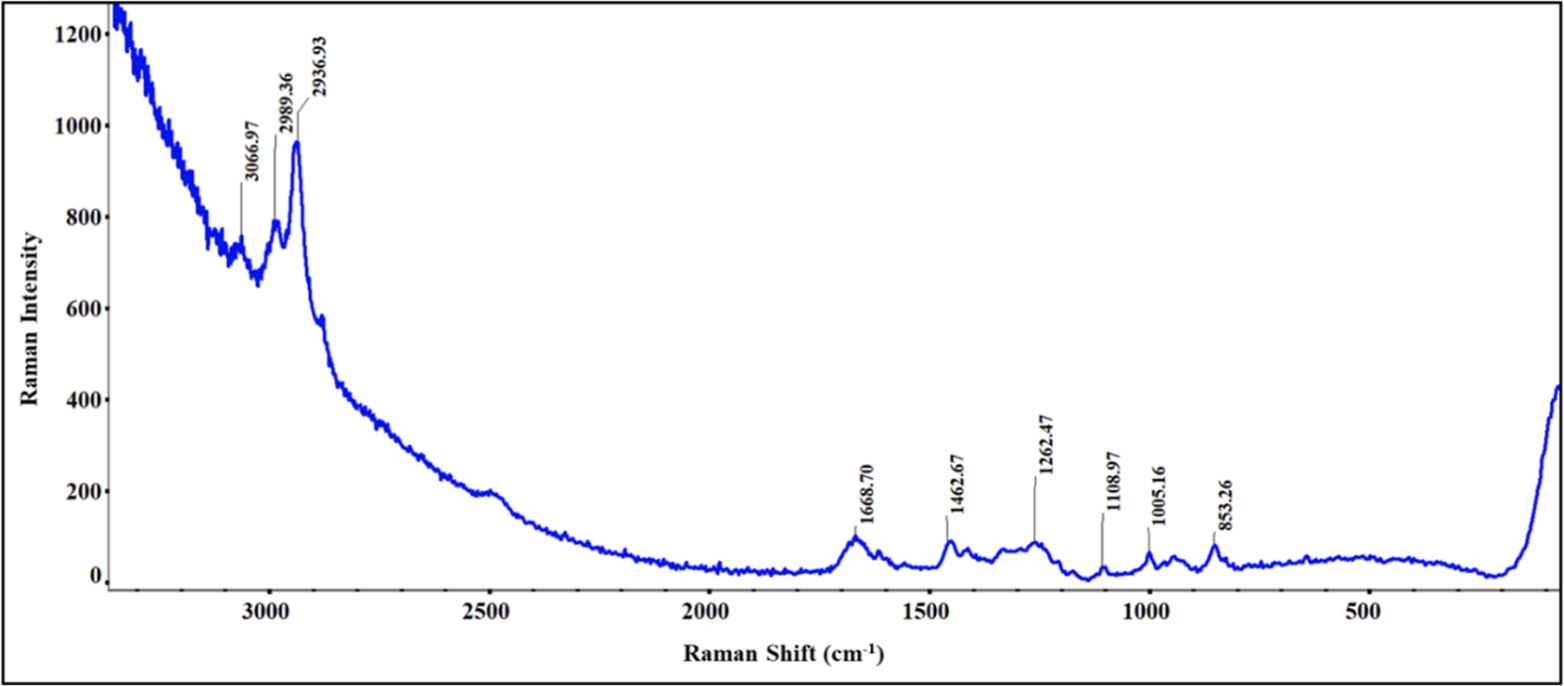
Representative Raman spectrum of raw SDP-4 powder showing characteristic Raman vibrational modes.

The Raman shifts observed in raw SDP-4 protein, attributed to various vibrational modes of its constituent amino acids and their organization in a secondary or tertiary structure, are shown in Figure 6. The shifts at 3066.97 cm^–1^ (aromatic C–H stretching), 1005.16 cm^–1^ (ring breathing mode of aromatic amino acids), and 853.26 cm^–1^ (C–H out-of-plane bending vibrations in aromatic rings) are indicative of the presence of tyrosine residues.^44^ The shifts at 2989.36 cm^–1^ and 2936.93 cm^–1^ correspond to C–H stretching vibrational modes in aliphatic chains, which are common in glycine, alanine, and serine.^44,45^ The amide I band at 1688.70 cm^–1^, primarily a C=O stretching vibrational mode in the protein backbone, is sensitive to the secondary structure of the protein. Similarly, the vibrational mode at 1262.47 cm^–1^, representing the amide III band due to C–N and N–H stretching vibrations, also provides information about the secondary structure of the protein.^46^ The shift at 1462.67 cm^–1^ is related to CH_2_ bending vibration mode, and the shift at 1108.97 cm^–1^ corresponds to the C–N stretching vibration mode in the protein backbone.^46^

### Raman Microscopy for Powder Mapping

**Figure 7:**
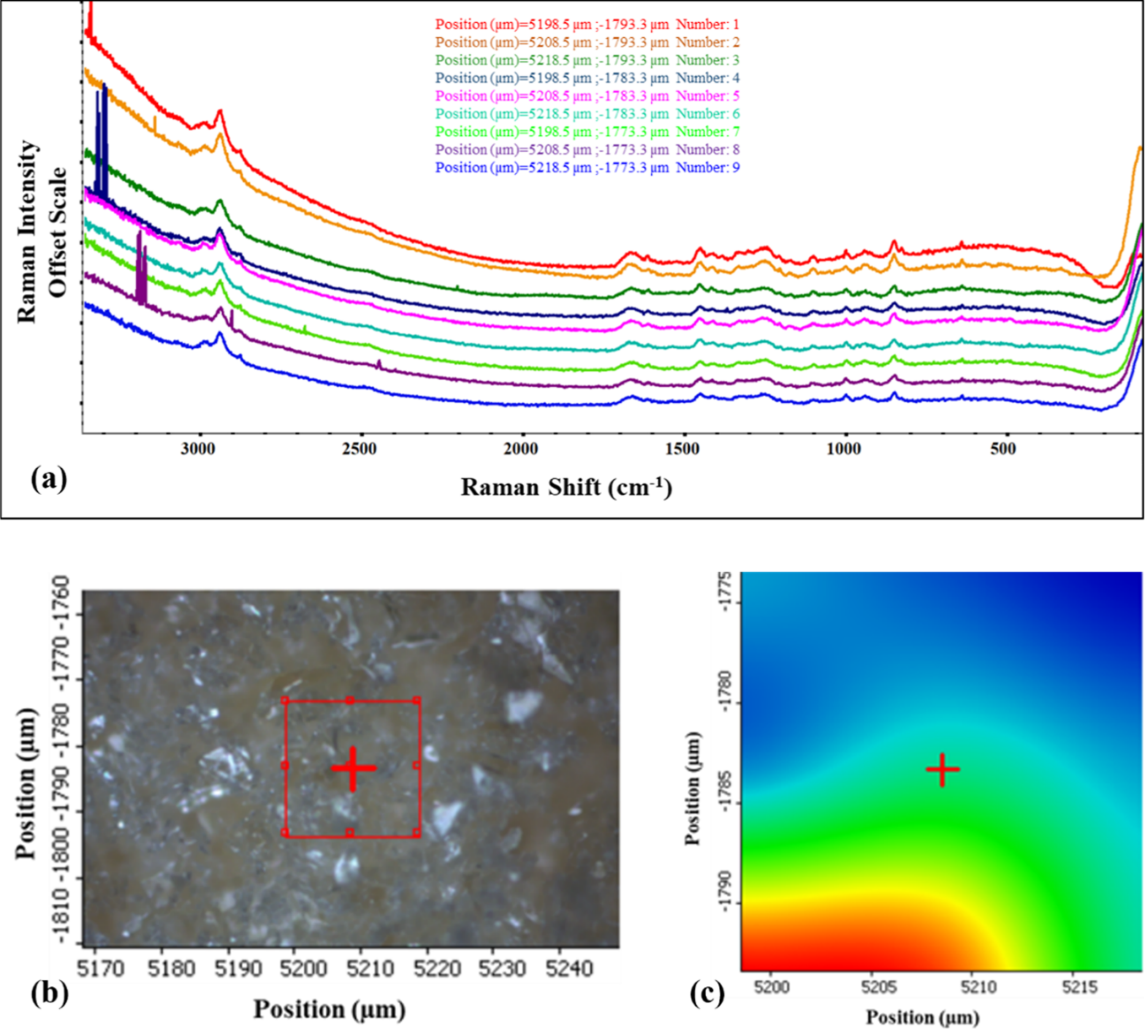
(a) Raman imaging map of raw SDP-4 powder, showing spectra collected from nine distinct sample points, with the *Y*-axis of each overlay map presented on an offset scale. (b) 2D Raman surface map captured under a 10× objective, highlighting the analyzed region with a red square. (c) 2D contour map of the highlighted red square region, illustrating the position coordinates for each spectrum. The spectra, acquired using a 785 nm laser, are color-coded based on their position coordinates.

Figure 7 shows the Raman chemical imaging map of raw SDP-4 powder derived from Raman spectra recorded at nine distinct coordinates. The spectra exhibit consistent patterns with no significant deviations, highlighting the uniformity of the protein sample. The spatial distribution analysis confirms of the homogeneity of the powder bed and absence of detectable impurities. The Raman map also reveals both broad and sharp peaks, indicative of amorphous and crystalline regions in the powder sample, thus confirming that SDP-4 is a semicrystalline substance.^47^ These findings align with the observations from the DSC and HSM analyses.

### ATR-FTIR Spectral Analysis

**Figure 8:**
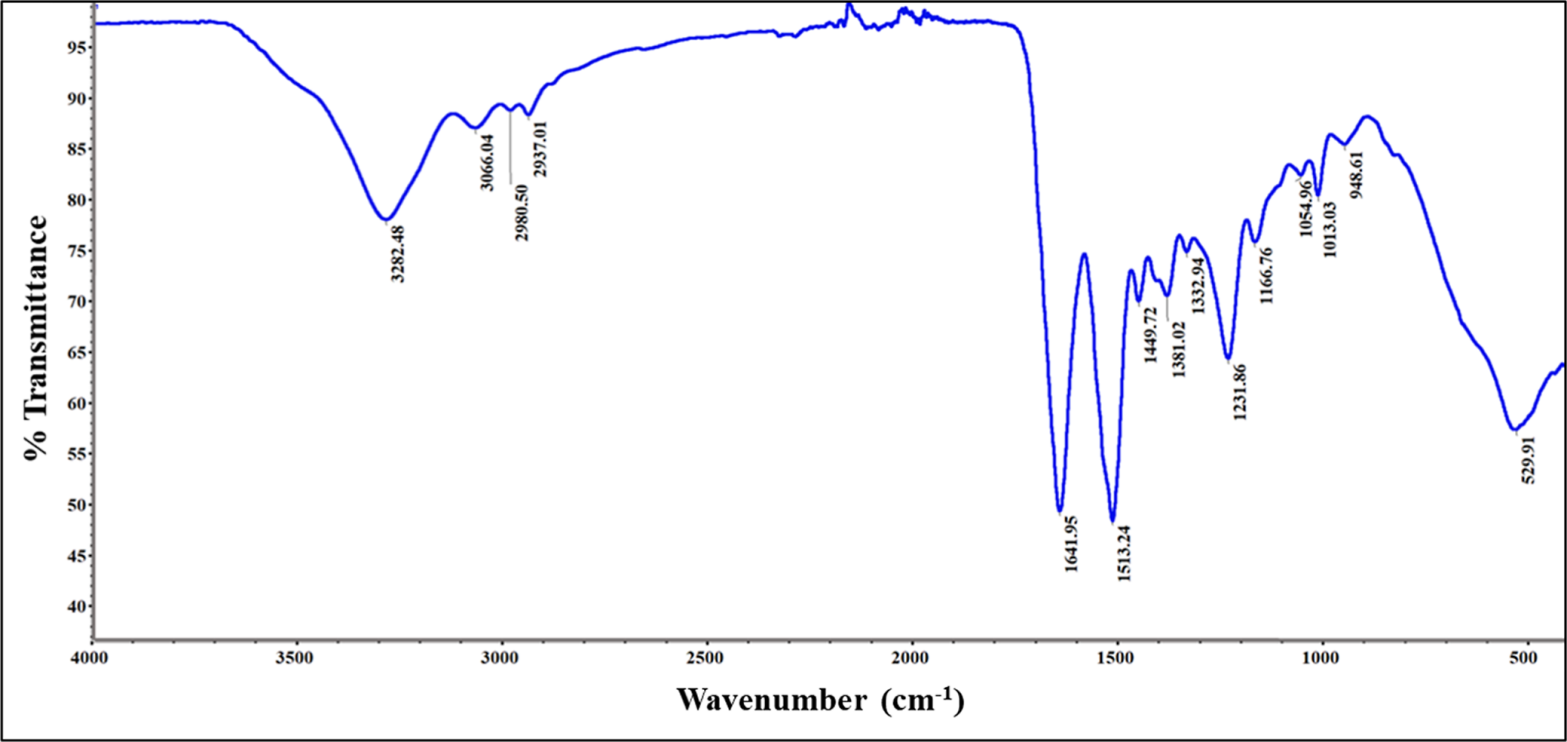
Representative ATR-FTIR-spectrum of raw SDP-4 powder showing characteristic molecular fingerprints.

Figure 8 presents the representative ATR-FTIR spectrum used for molecular fingerprinting of the raw SDP-4 powder. The spectrum reveals key information about its chemical structure. The peak at 3282.48 cm^–1^ is a characteristic N–H stretching vibration found in the amide group of the peptide bond. The peak at 3066.04 cm^–1^ is most likely from the stretching vibrations of the aromatic C–H bonds in tyrosine, one of the amino acid constituents of the protein.^48^ The peaks at 2980.50 and 2937.01 cm^–1^ correspond to C–H stretching vibrations of the aliphatic chains in the glycine, alanine, and serine residues.^49,50^ The characteristic 1641.95 cm^–1^ peak corresponds to C=O amide I band stretching vibrations in the protein backbone.^50^ These values are indicative of random coil secondary structures that appear in the range 1623–1641 cm^–1^. In addition, the lack of a distinctive shoulder at 1624 indicates that no appreciable amount of beta-sheets are present, as correlated with previous studies.^22,51^ Similarly, the peaks at 1513.24 and 1231.86 cm^–1^ may be associated with C–N stretching or N–H bending vibrations of amide II and III bands and provide more evidence of the prevalent random coil structure. The peak at 1641.95 cm^–1^ corresponds to the C–N stretching vibrations in the protein backbone. The peaks at 1449.72 and 1381.02 cm^–1^ correspond to CH_2_ and CH_3_ bending vibrations of the side chains of amino acids such as serine and alanine. Characteristics peaks at 1054.96, 1013.03, and 948.61 cm^–1^ correspond to C–O stretching, C–C in-phase stretching vibrations (ring breathing mode), and C–H out-of-plane bending vibrations in the aromatic tyrosine residue. A low-frequency peak at 529.91 cm^–1^ likely corresponds to residual phosphates from the native protein.^52^

### ATR-FTIR Microscopy for Powder Mapping

**Figure 9:**
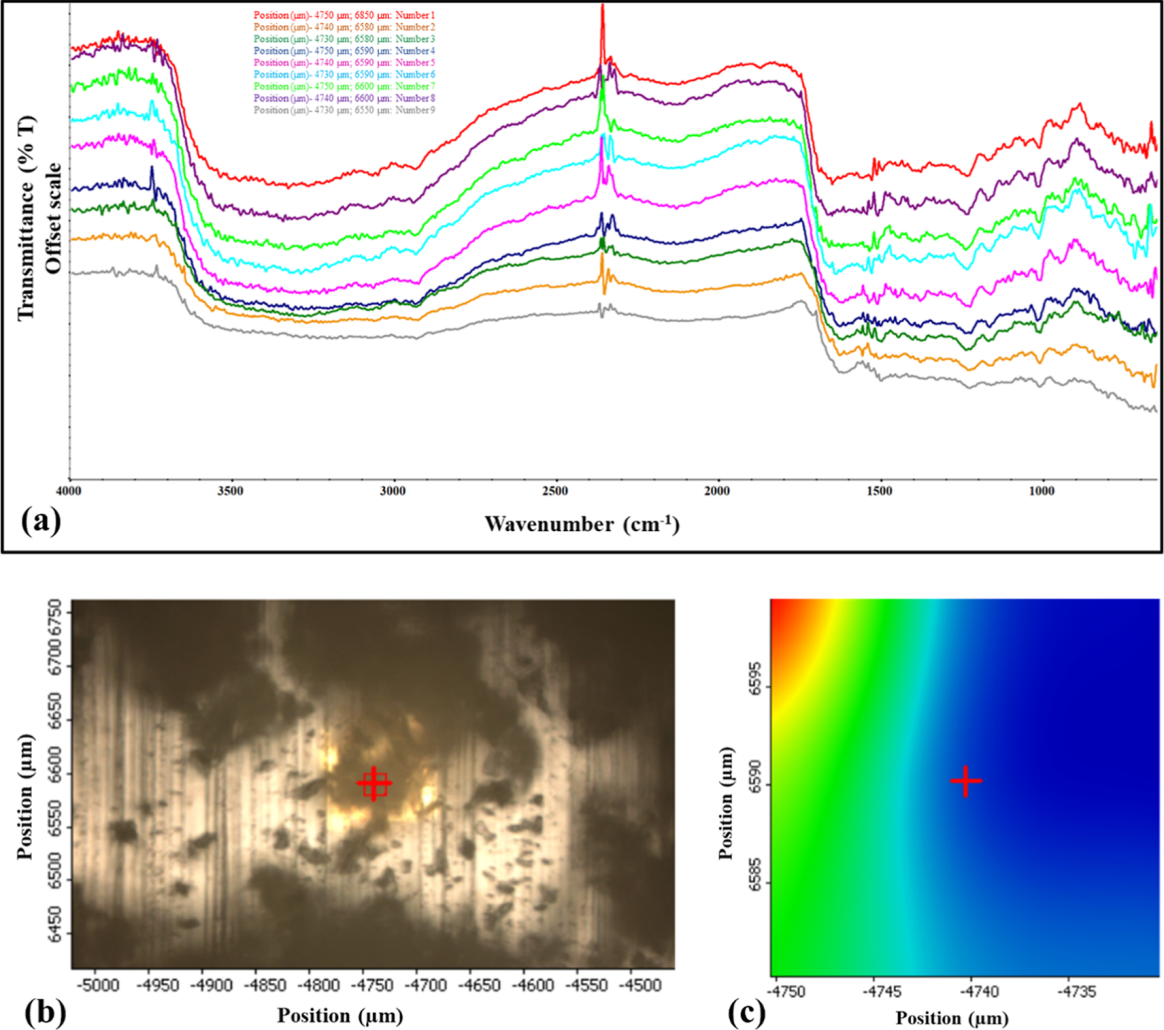
(a) FTIR overlay map of raw SDP-4 powders illustrating spectra collected from nine different locations on the sample, represented on an offset scale. (b) 2D surface map captured using a 10× objective, with a red square highlighting the analyzed region. (c) Contour map of the red square region, depicting the position coordinates of each spectrum. The overlay spectra represent are color-coded based on their position coordinates.

The FTIR microscopy chemical imaging map of raw SDP-4 powder is shown in Figure 9. The spectra overlay obtained by performing FTIR spectroscopy of nine distinct spots (position coordinates of spots shown in different colors in the overlay) shows spatial homogeneity, demonstrating the uniformity of the raw SDP-4 powder and the absence of any significant impurities.

### Residual Water Content by KFT

The raw SDP-4 powders had a residual water content of 9.61 ± 2.59% (w/w), which is above the recommended 5% limit for dry powder inhalers (DPIs). High moisture content may indicate a hygroscopic propensity of the SDP-4 material corresponding with the presence of polar amino acid functional groups from the presence of serine, threonine, and tyrosine that make up nearly 20% of amino acid content.^22^

### Water Vapor Isotherms by GVS

**Figure 10:**
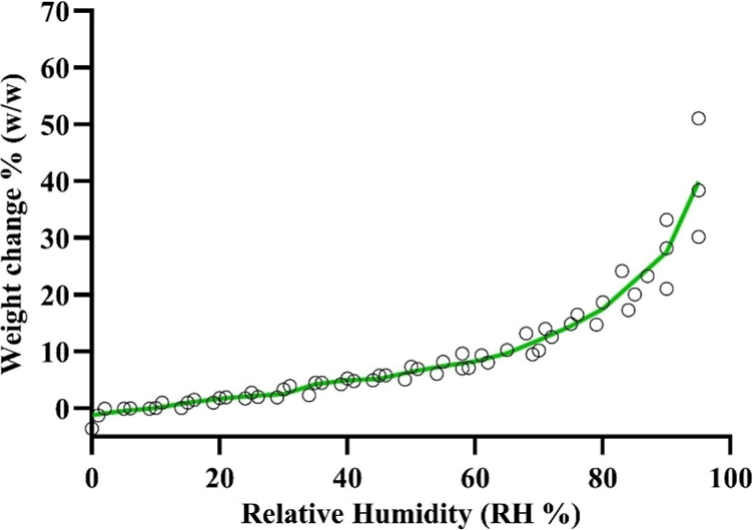
Gravimetric water vapor sorption isotherm of raw SDP-4 powder (weight change % vs RH) at 25 °C. The green line is the average of three individual experiments.

The vapor sorption isotherms in Figure 10 show the percentage weight change of the SDP-4 powder exposed to varying relative humidity levels at 25 °C. SDP-4 showed increased weight gain due to moisture absorption, particularly beyond 65% relative humidity (RH). At 95% RH, the weight gain reached nearly 40%, indicating significant water vapor uptake.

### Cell Viability as a Function of SDP-4 Dose

**Figure 11:**
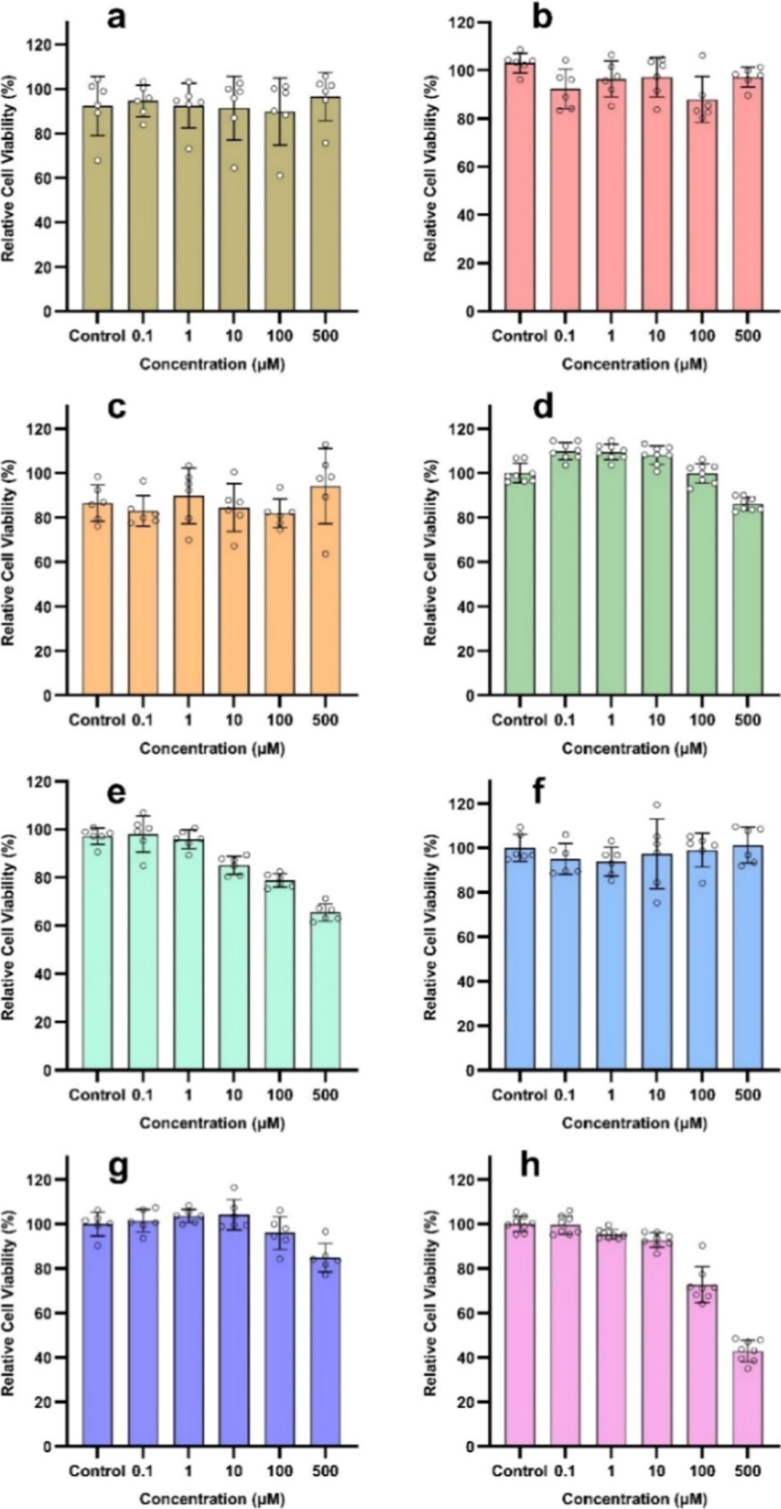
In vitro cell viability of raw SDP-4 powder in a dose-dependent manner across various cell lines: (a) A549 (mean ± SD, *N* = 6), (b) H358 (mean ± SD, *N* = 6), (c) Calu-3 (mean ± SD, *N* = 6), (d) HaCaT (mean ± SD, *N* = 8), (e) NHEK (mean ± SD, *N* = 6), (f) NHA (mean ± SD, *N* = 6), (g) RPMI 2650 (mean ± SD, *N* = 6), and (h) hCMEC/d3 (mean ± SD, *N* = 8) cell lines. Each bar represents the average value with standard deviation.

The relative cell viability (%) following 48 h of exposure to varying concentrations of raw SDP-4 powder on confluent monolayers of cells from various origins (lung, skin, nasal, and brain tissue) is shown in Figure 11. A wide dose range (up to 500 μM) demonstrated safety and excellent biocompatibility in all cells of respiratory, skin, nasal, and nervous tissue origin, except in human cerebral microvasculature endothelial cells, where dose safety was observed up to a concentration of 100 μM.

### Membrane Integrity and Tightness Using TEER Measurements

**Figure 12:**
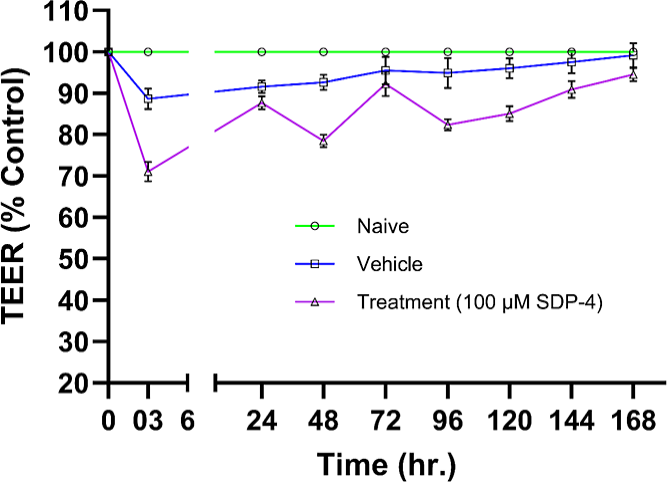
TEER values (% of control) measured at different time intervals using an STX4 open-end electrode connected to an EVOM TEER meter. The TEER measurements were performed across HaCaT cells in Transwells under ALI conditions following treatment with 100 μM SDP-4, as well as for vehicle-treated and untreated (naïve) controls. Data are presented as mean ± SD from *n* = 4 replicates. Statistical significance was determined using two-way ANOVA with Tukey’s posthoc test (*p* < 0.05).

Figure 12 displays the TEER values (% of control) measured at 3 h and subsequently over a period of 7 days following treatment with 100 μM SDP-4, vehicle, or untreated (naïve) conditions. Treatment with 100 μM SDP-4 caused a transient drop in TEER values to approximately 70% of control after 3 h, followed by partial recovery to 87% at 24 h. However, TEER values fluctuated over the next 3 days before returning to control levels after 7 days. In contrast, vehicle treatment resulted in a smaller transient drop in TEER values, which quickly returned to baseline. A two-way ANOVA followed by Tukey’s multiple comparison test (*p* < 0.05) was performed to compare the means between different time points across rows and columns. Statistically significant differences in TEER values were noted 3 h post-treatment, with the vehicle (*p* = 0.005) and SDP-4 (*p* = 0.0003) compared to the naïve conditions. No significant differences were noted after 7 days when comparing SDP-4 treatment to naïve conditions. However, vehicle-treated cells returned to naïve conditions earlier, within 24 h, with statistical insignificance observed only after 3 days. Comparing the early recovery (3 h) of the vehicle to that of SDP-4 treatment revealed a statistically significant difference (*p* = 0.0001), indicating that SDP-4 treatment affected membrane integrity and barrier tightness. Additionally, fluctuations in membrane recovery after SDP-4 treatment were noted, compared to the more stable recovery observed with the vehicle, with no significant differences at 24 and 72 h.

## Discussion

The current study provides a comprehensive characterization of SDP-4, a soluble fibroin protein hydrolysate derived from silkworm cocoons, highlighting its unique physicochemical properties and biocompatibility. SDP-4 has been developed as a first-in-class biodegradable and biocompatible biopolymer and is currently being used as wetting agent in commercial eye drop products for treating ocular surface disease. As far as we are aware, this is the first study to offer comprehensive insights into physicochemical characterizations, dose-dependent cell viability across different human cells, and membrane integrity studies, which have not been reported previously. Given its therapeutic potential, understanding the detailed structural, thermal, and chemical properties of SDP-4 is crucial for optimizing its formulation and application.

SEM morphological analysis (Figure 2) reveals a glassy flake morphology with a wide particle size distribution. SEM coupled to EDX (Figure 3) verified the elemental constituents as expected in the backbone structure of the protein. The presence of trace processing impurities like Na, Cl, and P are within the pharmaceutical limits, and the absence of any heavy metals supports that the lyophilized protein is of high purity, suitable for therapeutic use. Various studies have reported that silk fibroin derived from the cocoons of *Bombyx mori* exhibits different morphologies, ranging from irregular spherical and filamentous to flaky forms, depending on the specific extraction conditions and solvents used for regeneration.^53−55^

SDP-4 differs from native silk fibroin protein in its total amino acid composition, cysteine disulfide bonds between the heavy and light fibroin chains, serine content and molecular weight.^26,27^ These compositional and covalent linkage changes in SDP-4 compared to native silk fibroin protein led to alterations in the hierarchical organization of the protein’s secondary, tertiary, and quaternary structures. The appearance, wide size distribution, and molecular weight distribution of SDP-4 depend on the processing conditions, such as the solvents used during the regeneration process. Native silk fibroin is a semicrystalline protein,^56^ whereas SDP-4 has a reduced β- sheet activity, making it more soluble than its congener.^26^ Other factors contributing to the glassy flake appearance of SDP- 4 may include chain folding and cross-linking between the native fibroin chains.

DSC thermograms of raw SDP-4 powder, illustrated in Figure 4, and main thermodynamic transitions (Table 1), reveal key thermal properties of the protein. The fourth peak, with an enthalpy of 49.17 ± 3.55 J/g at a peak temperature of 216.79 ± 10.83 °C, represents the main thermal transition of the raw SDP-4 powder. This first-order transition refers to the change of phase from solid to liquid (melting point). The glass transition temperature (*T*_g_), indicating the transition of an amorphous material (or amorphous regions within a semicrystalline material) from a hard, glassy to a rubbery state, was calculated using the inflection midpoint method. The *T*_g_, seen as a step change in the baseline of the DSC heat flow curve, was found to be 103.54 ± 1.23 °C. The higher *T*_g_ value above 100 °C suggests that the protein can withstand higher temperatures before undergoing significant structural changes. Formulations of proteins with high *T*_g_ also suggest long-term stability during storage, which has significant implications for the shelf life of the product.^57^ The minor second and third peaks, with distinctly low enthalpy values, may be due to changes in the conformational flexibility of the protein, such as the loss of quaternary and tertiary structures associated with weak forces of attraction. Multiple thermal transitions are present after the first-order transition, and it is clear that these transitions are staggered together. The reasons for these transitions may be attributed to various factors, such as the disruption of secondary structure and the loss of disulfide bonds. The staggered nature of these transitions as a function of temperature represents structural changes in the organization of the protein structure, stabilized by different types of interactions, such as hydrogen bonding, hydrophobic interactions, and ionic interactions.^58,59^

The HSM images of SDP-4 observed at different temperatures (Figure 5) offer valuable information on the thermal behavior and structural characteristics of the protein. Below its melting point (216.79 ± 10.83 °C), the protein exhibits birefringence, indicating its semicrystalline nature. Birefringence is a key feature of materials with ordered structures, such as crystals, where different parts of the material refract light at different angles. At temperatures exceeding the melting temperature, loss of birefringence signifies the breakdown of the crystalline structure, likely due to thermal degradation. The transition from a semicrystalline to an amorphous state and subsequent degradation at elevated temperatures has important implications for the stability and application of SDP-4 in various formulations. The maintenance of birefringence up to the melting point suggests that the protein retains some degree of structural integrity, which could be beneficial for maintaining its functional properties, especially when developing stable formulations for pharmaceutical or biomedical applications.

The structural and chemical characteristics of the raw SDP-4 protein were extensively analyzed by using Raman (Figure 6) and FTIR spectroscopy (Figure 8). Both techniques revealed the presence of key amino acid residues and structural motifs. Raman spectroscopy identified significant vibrational modes, including aromatic and aliphatic C–H stretches and amide bands, indicating the protein’s secondary structure and confirming the presence of tyrosine, glycine, alanine, and serine. The uniformity in the Raman chemical imaging map (Figure 7) suggests a consistent composition and the presence of both amorphous and crystalline regions, indicative of a semicrystalline nature. FTIR spectroscopy complemented these findings by highlighting characteristic peaks associated with peptide bonds, aromatic structures, and secondary structure components. Peaks such as the amide I band and those related to CH_2_ and CH_3_ bending vibrations provided further details on the secondary structure and chemical composition. The FTIR chemical imaging map (Figure 9) reinforced the uniformity and purity of the SDP-4 powder, confirming its homogeneity and suitability for pharmaceutical applications. Together, these spectroscopic analyses elucidate the structural integrity and uniformity of SDP-4, critical for its stability and effectiveness as a therapeutic agent.

The residual water content of raw SDP-4 powders, quantified by KFT (Table 2), was found to be 9.61 ± 2.59% (w/w). Since the powder is being studied as a prospective candidate for dry powder inhalation, the residual water content exceeds the guideline of 5% (w/w) for a dry powder inhaler (DPI).^60^ Excess residual water content can lead to increased hygroscopicity; high residual water content can cause particle agglomeration, impairing aerosolization performance and flow properties. In addition to the high residual water content, the particle size of these powders is also not suitable for aerosolization. Therefore, for the preparation of a DPI of this protein, drying methods must be optimized to achieve the desired particle size and moisture content. Appropriate moisture barrier packaging is essential to prevent exposure to environmental humidity, and stability tests must be performed to ensure the DPI remains stable during its shelf life.^61^

**Table 2:** Residual Water Content (*n* = 3) of Raw SDP-4 Powders Calculated by KFT

raw SDP-4 samples	water content (% w/w)
SDP-4 *n* = 1	6.909
SDP-4 *n* = 2	12.084
SDP-4 *n* = 3	9.805
average ± SD	9.599 ± 2.594

In the vapor sorption isotherms (Figure 10), the percentage weight change of raw SDP-4 powders at 25 °C reveals that the material’s moisture uptake increases progressively with rising relative humidity. As illustrated, the powder steadily absorbs moisture up to an RH of 65%. Beyond this point, the weight gain increases steeply, reaching a near 40% increase at 95% RH. At 75% RH, which is the RH used in accelerated stability conditions, the powder gains an additional 15% of its weight due to excessive water vapor uptake. The isotherm follows the BET (Brunauer–Emmett–Teller) Class II isotherm, which is sigmoidal. Langmuir monolayer water vapor adsorption is known to occur in the very low RH range 0–5%, followed by multilayer BET sorption. The plateau region represents a lyotropic phase transition over a distinct finite RH range. After the lyotropic phase transition, significant water vapor uptake increases sharply with increasing RH at 85% and higher with no plateau. This behavior demonstrates that SDP-4 has a natural tendency to be hygroscopic. The high surface area due to the sample’s flake-like morphology and the presence of hydrophilic groups in the protein further contributes to high-water vapor uptake. As a prospective candidate for DPI formulation, it is crucial to reduce the additional moisture to prevent the powders from becoming cohesive and less free-flowing, which can negatively impact their aerodynamic properties. For successful DPI formulation, careful consideration must be given to particle formation and drying methods that minimize moisture content. For instance, the protein can be spray-dried in a closed-loop, nonaqueous mode using organic solvents at temperatures below its transition state. Particle engineering techniques such as spray drying to produce spherical shapes with minimal surface area can improve aerodynamic properties. Additionally, cospray drying with excipients such as mannitol, trehalose, or lactose can reduce moisture absorption, improve flow properties, and enhance the stability of the protein. Moisture-resistant packaging materials and desiccants should also be used in packaging to ensure product stability.

Silk-derived proteins are emerging as promising candidates for commercial applications across various industries, including medical, biomedical, cosmetic, pharmaceutical, and food industries.^62−67^ SDP-4 powder demonstrated safety and excellent biocompatibility across various cell types (lung, skin, nasal, and nervous tissue) at concentrations of up to 500 μM (Figure 11). Dose-dependent viability assays were conducted on two different human keratinocyte cell lines, HaCaT and NHEK. SDP-4 demonstrated over 80% cell viability in HaCaT cells at concentrations up to 500 μM, while NHEK cells showed only 65% viability at the same dose. These findings may highlight the resilient nature and distinct microenvironmental conditions of immortalized HaCaT cells compared to primary NHEK cell lines.

Recent studies have further indicated that SDP-4 has dose-dependent efficacy in inhibiting NF-kB-driven gene expression of TNF-α and MMP-9, which are known to be critical mediators in the progression of lung diseases such as chronic obstructive pulmonary disease (COPD), acute lung injury (ALI), acute respiratory distress syndrome (ARDS), asthma, and pulmonary fibrosis.^22,68−70^ Additionally, these inflammatory mediators are significant in tuberculosis pathophysiology, influencing immune cell recruitment, granuloma formation, tissue remodeling, and tissue destruction.^71,72^ In exploring SDP-4 as a potential therapeutic protein for lung diseases, we evaluated its biocompatibility and dose-dependent cell viability across various respiratory-origin cell lines including human lung epithelial cells (A549 and H358, representing distal regions) and Calu-3 cells (representing proximal regions of the respiratory tract). All of these cell lines showed over 80% viability at SDP-4 concentrations as high as 500 μM. Additionally, we tested the safety margin of SDP-4 in human nasal epithelial cells (RPMI 2650), finding over 80% viability at 500 μM, indicating potential safety for nasal formulations targeting respiratory diseases. Furthermore, biocompatibility assessments were conducted on normal human astrocytes (NHA) and human cerebral microvasculature endothelial cells (hcMEC/d3), which represent central nervous system cells and the blood–brain barrier, respectively.^73^ Cell viability of more than 100% can be noted at specific doses in Figure 11b,d,g, representing the cell lines H358, HaCaT, and RPMI-2650. Statistical analysis using one-way ANOVA reveals that cell viability above 100% (in HaCaT cells) is only significant when control is compared to 0.1 and 1.0 μM doses using a Dunnett’s post comparison test with *p*-values of 0.027 and 0.025 at an α of 0.05. The more than 100% cell viability seen in H358 and RPMI-2650 is statistically insignificant. Cell viability greater than 100% also can indicate regeneration, which can be an advantageous effect.

Silk fibroin-based materials are known for their safety and ability to promote cell migration, proliferation, and differentiation in NHA cells. Consistent with these properties, SDP-4 exhibited excellent biocompatibility in NHA cells with close to 100% viability at 500 μM. However, hcMEC/d3 cells dosed with the maximal 500 μM concentration of SDP-4 demonstrated only 42% viability, which increased to 72% viability at a lower 100 μM dosing concentration, suggesting that higher doses may be harmful due to the unique characteristics of these cells. As part of the blood-brain barrier (BBB), these cells have specialized functions and structures that may make them more vulnerable to certain compounds.^74,75^ Overall, these results underscore that SDP-4 has a favorable safety margin in vitro. While a therapeutic index of 10 μM is considered robust for protein therapeutics such as antibodies, there is no universally acceptable threshold for protein therapeutics. Regulatory guidelines do not prescribe a single concentration standard for therapeutic proteins, as safe and effective doses depend on the specific disease, the protein’s properties, and patient factors. Comprehensive preclinical and clinical testing is essential to determine the appropriate dosage for any intended patient population.

To illustrate the dose-dependent effects on in vitro cell viability, both linear and nonlinear regression analyses were performed to best characterize the correlation between the SDP-4 dose and cell viability. Regression analyses of cell viability (Figure 11a–h) revealed cell-specific responses to SDP-4. In A549, H358, Calu-3, and NHA cells (Figure 11a,–c,f), correlation coefficients from linear and nonlinear analyses indicated no correlation, suggesting that SDP-4 doses up to 500 μM have a minimal impact on viability and are thus considered safe for these cell types. In RPMI-2650 cells (Figure 11g), a moderate correlation was observed in both linear and nonlinear models, indicating a minor decline in viability as the dose increased. However, in HaCaT and NHEK cells (Figure 11d,e), moderate to strong correlations were observed with linear regression, indicating downward trends in viability at much higher doses. Notably, in hCMEC/D3 cells (Figure 11h), strong correlations were seen in both linear and nonlinear regression models with high correlation coefficients (0.901 and 0.927), suggesting a clear dose–response relationship. Here, 500 μM SDP-4 reduced cell viability below 50%, suggesting potential toxicity near this concentration.

To evaluate the effect of SDP- 4 on human membrane integrity, TEER values (Figure 12) were measured at 3 h post-treatment and subsequently over a 7 day period following exposure to 100 μM SDP-4, vehicle, or untreated (naïve) conditions. The data show a significant reduction in TEER values, dropping to approximately 70% after 3 h of SDP-4 treatment, followed by a recovery to 87% after 24 h. This recovery, however, was transient, as TEER values fluctuated over the next 3 days (48, 72, and 96 h) between 78%, 92%, and 82%, respectively. After 96 h, the TEER values gradually returned to the levels observed under naïve conditions (air–liquid interface conditions) after 168 h (7 days). In comparison, the vehicle used to solubilize SDP-4 protein (nonsupplemental EMEM) caused a transient drop to 88% after 3 h of treatment, followed by a steep recovery to naïve conditions.

HaCaT cells are immortalized dermal keratinocytes and form tight junctions, resulting in a blockade of electric signal across their monolayers, and under normal physiology, this resistance can be measured. We studied the membrane integrity and recovery of the barrier tightness upon treatment with a solubilized protein hydrolysate, SDP-4, by measuring the electric resistance across the surface of a Transwell membrane system. Statistical analysis revealed significant differences in TEER values at 3 h between SDP-4 and vehicle treatments, with SDP-4 showing greater disruption. Despite initial fluctuations and temporary permeability changes, SDP-4-treated cells ultimately restored barrier integrity, suggesting that while SDP-4 may temporarily disrupt membrane tightness, cells can repair the barrier over time. The fluctuations in the TEER values upon treatment with SDP-4 under ALI conditions could be attributed to several factors. SDP-4 may interact with cell membranes in a manner that temporarily disrupts tight junctions, leading to TEER fluctuations, followed by a recovery phase as cells adapt or repair the junctions. Other potential factors include protein aggregation over time and its interactions with the membrane layer or tight junctions or a fluctuating stress response and recovery to the protein. These findings suggest that SDP-4 may transiently increase membrane permeability or disrupt junctions to facilitate its transport.^76^ However, the eventual recovery indicates that the cells can repair the barrier over time. Understanding these dynamics is crucial for evaluating the therapeutic potential of SDP-4, especially concerning skin barrier functions, dose safety, and drug delivery.

The results presented herein demonstrate SDP-4’s distinct morphological features, chemical uniformity, and stability under varying environmental conditions. Moreover, the biocompatibility studies underscore its safety across a range of human cell types with observed dose-dependent responses and membrane interactions. These findings not only confirm the suitability of SDP-4 for pharmaceutical applications but also pave the way for further exploration of its potential uses in treating respiratory diseases and other diseases.

## Conclusions

In this study, for the first time, we comprehensively characterized SDP-4-lyophilized powder using various analytical techniques, microscopy, in vitro TEER, and in vitro cell viability across different human cell types as a function of dose. The unique amino acid composition and reduced β-sheet content of SDP-4 contribute to its glassy flake appearance. Thermal analysis revealed a very stable first-order transition and a high glass transition temperature, indicating stability under elevated temperatures, which is critical for storage and formulation purposes. Elemental analysis and spectroscopic techniques confirmed the presence of structural constituents and a semicrystalline nature, with both amorphous and crystalline regions.

In vitro biocompatibility assessments showed that SDP-4 maintains a favorable safety margin across various human cell lines, suggesting its potential for vast therapeutic applications. However, the observed toxicity in human cerebral microvasculature endothelial cells underscores the need for cautious dose optimization. The transient fluctuations in TEER values indicate that SDP-4 may temporarily mildly disrupt cell membranes, potentially facilitating its transport across biomembrane barriers, followed by membrane recovery. The percentage of fluctuation and phase recovery compared to the naïve conditions suggests these transitions are minor and necessary for cellular adaptation and repair. These comprehensive findings on SDP-4 suggest promising, vast applications in lung, skin, and brain delivery, where its unique properties could be beneficial.

## Data Availability

The data can be made available upon request from the Corresponding Author.

## Notes

Ethical approval statement: This article does not include any studies involving human participants or animals conducted by any of the authors.
